# Role of a Porcine Herpesvirus, PCMV/PRV, in Xenotransplantation

**DOI:** 10.3389/ti.2025.14087

**Published:** 2025-02-04

**Authors:** Joachim Denner

**Affiliations:** Institute of Virology, Free University Berlin, Berlin, Germany

**Keywords:** orthotopic pig heart transplantation, porcine cytomegalovirus/porcine roseolovirus, virus safety, xenotransplantation, survival time

## Introduction

Xenotransplantation using pig organs may be associated with the transmission of porcine viruses that could cause disease in recipients. A well-known example is the porcine cytomegalovirus, which is actually a porcine roseolovirus, hence abbreviated as PCMV/PRV. This virus is related to human herpesviruses 6 and 7 and is not closely related to human cytomegalovirus, which causes significant complications in allotransplantation [1]. PCMV/PRV has been shown to drastically reduce the survival time of porcine organs in non-human primates (for review, see [2]). The virus was also transmitted to the first patient in Baltimore who received a pig heart; it replicated exponentially to high titers in the transplanted pig heart and likely contributed to the patient’s death [3]. Therefore, the transmission of PCMV/PRV and other potentially zoonotic porcine viruses should be prevented.

Längin et al. highlighted the progress in orthotopic pig heart transplantation in non-human primates [4]. Since the first study in 1994, it has been possible to increase the survival time of orthotopically transplanted pig hearts from 39 to 59 to 195 and finally to 264 days. In addition to advancements in multiple genetically modified donor pigs, organ preservation, new immunosuppressive and immunomodulatory drugs, and growth inhibition of the transplanted organ, the authors discussed the virological safety of xenotransplantation. Unfortunately, in this context, Längin et al. [4] cited an abstract from the International Xenotransplantation Association Conference in San Diego in 2023 without critical commentary. In the abstract, Zhang et al. [5] claimed that their investigations found no difference in survival times of pig heart transplants from PCMV/PRV-positive versus PCMV/PRV-negative donor animals in baboons. In these 12 donor pigs, PCMV/PRV was tested only by PCR; six animals (50%) were positive, but no differences in transplant or recipient survival were observed [6]. This study warrants critical scrutiny because it contradicts all previous findings and could lead to an underestimation of the risks posed by PCMV/PRV.

## The Risk Posed by PCMV/PRV

As reported as early as 2014, PCMV/PRV significantly reduced the survival times of pig kidneys transplanted into baboons and cynomolgus monkeys [6, 7]. Kidneys infected with PCMV/PRV survived no longer than 14 days, whereas virus-free organs survived up to 53 days. Similarly, the absence of PCMV/PRV was a key factor in prolonging the survival time of orthotopic pig heart transplants in baboons: pig hearts infected with PCMV/PRV never lasted beyond 30 days, while virus-free transplants survived up to 195 days [8].

How, then, can the findings of Zhang et al. [5] be explained? False-negative PCR results may occur when the virus is no longer detectable in tested samples because it has entered latency, a hallmark of herpesviruses like PCMV/PRV [9]. Conversely, false-positive PCR results - such as the one from the donor animal whose recipient survived 225 days - are harder to interpret and are most likely due to contamination during PCR. Unfortunately, the PCR methodology was not described in detail in the abstract. Retesting could help resolve the discrepancies between Zhang et al.’s results [5] and previously published data [2, 6–8]. Additional immunological screening for antibodies against PCMV/PRV in donor pigs - a preferred method for detecting latent PCMV/PRV infection [9] - or testing recipient baboons for PCMV/PRV, as the virus should be present in all organs even after short survival times as shown by us [10], could also provide clarity. We would be happy to offer our expertise and methodologies to support these investigations.

## Data Availability

The original contributions presented in the study are included in the article/supplementary material, further inquiries can be directed to the corresponding author.
